# Supplementary figures and images for: Transcriptome analysis of peripheral blood of Schistosoma mansoni infected children from the Albert Nile region in Uganda reveals genes implicated in fibrosis pathology
Source: PLoS Negl Trop Dis. 2023 Nov 15;17(11):e0011455. doi: 10.1371/journal.pntd.0011455 (PMC10686515; doi:10.1371/journal.pntd.0011455)

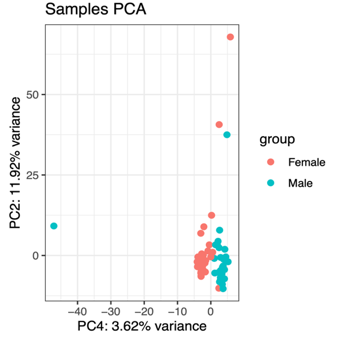

Supplement: S1 Fig — (TIF) [file pntd.0011455.s001.tif]

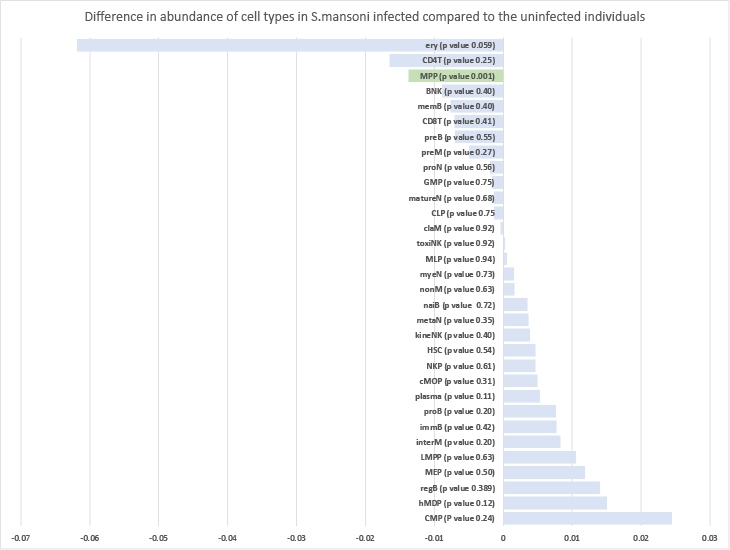

Supplement: S2 Fig — (TIF) [file pntd.0011455.s002.tif]
