# Supplementary material for: Transcriptome analysis of peripheral blood of Schistosoma mansoni infected children from the Albert Nile region in Uganda reveals genes implicated in fibrosis pathology
Source: PLoS Negl Trop Dis. 2023 Nov 15;17(11):e0011455. doi: 10.1371/journal.pntd.0011455 (PMC10686515; doi:10.1371/journal.pntd.0011455)
Supplement: S1 Table — (PDF) [file pntd.0011455.s003.pdf]

S1 Table: Table showing participant details for the RNAseq analysis

Sample	Sex	Site	Age	Height (cm)	Weight (Kg)	MUAC	HAZ	BAZ	POC-CCA	KK	CAA(pg/ml)	Classification
PS722T	Male	Dei	11	131	32	20	-1.8	0.8	3	NA	934616.84	High
PS957T	Male	Kayonga	10	125	28	29	-2.01	0.76	3	15	206112.64	High
PS349T	Female	Nyakagei	13	142	31	19.5	-2.07	-1.71	4	21.5	69301.61	High
PS336T	Male	Nyakagei	12	132	31	22.5	0	0	4	NA	21662.75	High
PS400T	Male	Nyakagei	13	144	35	20	-1.62	-0.69	1	9	21471.1	High
PS345T	Male	Nyakagei	10	116	21	16.5	-3.42	-0.52	4	17	20656.38	High
PS219T	Male	Kivuje	13	130	30	19	-3.51	-0.23	4	19.5	19675.41	High
PS773T	Male	Dei	15	151	37	19	-1.55	-1.79	4	NA	15833.65	High
PS361T	Female	Nyakagei	12	135	25	19	-2.37	-2.52	4	NA	15557.48	High
PS409T	Male	Nyakagei	12	139	36	21	-1.42	0.5	4	57	14722.97	High
PS303T	Female	Nyakagei	15	152	50	27	-1.41	0.47	4	19.5	12979.69	High
PS378T	Male	Nyakagei	11	132	30	18.6	-1.65	0.15	4	93.5	12606.67	High
PS357T	Female	Nyakagei	12	139	30	19.5	-1.79	-1.25	4	15.5	12012.35	High
PS329T	Male	Nyakagei	14	137	35	21.5	-3.4	-0.16	4	21.5	11612.61	High
PS949T	Female	Kayonga	14	135	38	23	-3.57	0.45	4	20.5	11410.18	High
PS189T	Male	Kivuje	14	139	41	23	-3.14	0.83	4	34	11238.1	High
PS1016T	Male	Kayonga	12	131	26	19	-2.55	-1.45	3	0	11049.34	High
PS343T	Male	Nyakagei	10	124	21	16	-2.16	-2.07	4	37	10968.56	High
PS711T	Female	Dei	10	130	25	16.5	-1.35	-1.03	3	52	10000	High
PS746T	Female	Dei	10	120	21	15	-2.91	-1.17	3	NA	10000	High
PS333T	Female	Nyakagei	12	128	25	19	-3.4	-1.41	4	2.5	9901.65	High
PS312T	Female	Nyakagei	14	151	42	22	-1.27	-0.46	4	8	9605.37	High
PS305T	Female	Nyakagei	15	152	46	23	-1.41	-0.11	4	6.5	9300.68	High
PS359T	Male	Nyakagei	11	133	30	19.2	-1.5	0.01	4	24	8556.76	High

PS348T	Female	Nyakagei	11	140	34	21.3	-0.75	0.05	4	18.5	6996.23	High
PS304T	Female	Nyakagei	15	154	51	25	-1.11	0.43	1	2.5	6901.97	High
PS220T	Male	Kivuje	13	133	28	19	-3.1	-1.34	4	60.5	5001.64	High
PS729T	Female	Dei	13	146	40	22.4	-1.49	-0.01	4	9.5	3371.01	Low
PS193T	Female	Kivuje	14	145	37	20	-2.13	-0.83	1	0.5	2156.6	Low
PS687T	Male	Dei	10	116	23	17	-3.42	0.35	3	7.5	2103.52	Low
PS256T	Female	Kivuje	12	142	33	19	-1.35	-0.77	1	6.5	1818.55	Low
PS373T	Female	Nyakagei	10	129	24	17.8	-1.51	-1.28	1	1	1629.94	Low
PS951T	Male	Kayonga	12	124	32	22	-3.54	1.28	1	1.5	1422.64	Low
PS707T	Female	Dei	13	151	38	20.1	-0.77	-0.97	1	NA	1152.97	Low
PS956T	Male	Kayonga	12	144	36	20	-0.72	-0.09	1	0	830.32	Low
PS277T	Female	Kivuje	12	136	32	20	-2.23	-0.31	1	4.5	522.01	Low
PS758T	Female	Dei	10	119	23	17.4	-3.07	-0.19	1	0	367.29	Low
PS168T	Female	Kivuje	15	156	45	23	-0.82	-0.67	1	0	357.84	Low
PS767T	Male	Dei	11	130	28	17.5	-1.95	-0.2	1	NA	295.05	Low
PS272T	Male	Kivuje	11	129	25	16	-2.1	-1.21	1	0	271.52	Low
PS702T	Female	Dei	12	146	43	24.3	-0.76	0.8	1	NA	241.21	Low
PS764T	Male	Dei	11	126	26	18.2	-2.54	-0.31	0.5	1	167.67	Low
PS246T	Female	Kivuje	10	124	26	19	-2.29	0.14	1	0	142.71	Low
PS223C	Male	Kivuje	10	118	27	19	-3.1	1.36	0	0	127.47	Low
PS353T	Male	Nyakagei	12	136	28	18.5	-1.85	-1.46	1	0	20.05	Neg
PS534C	Female	Alwi	14	152	40	21	-1.12	-0.96	0	0	13.32	Neg
PS513C	Male	Alwi	12	150	32	22	0.13	-2.2	0	0	11.9	Neg
PS750T	Male	Dei	12	140	32	17.5	-1.28	-0.66	0.5	NA	11.38	Neg
PS417T	Male	Nyakagei	10	123	23	16.5	-2.32	-0.8	1	0	10.95	Neg
PS506C	Male	Alwi	11	136	31	21	-1.06	-0.1	0	0	9.09	Neg
PS544C	Male	Alwi	11	122	21	18	-3.14	-1.95	0	0	7.79	Neg
PS941T	Male	Kayonga	10	122	26	21	-2.48	0.54	0.5	0	7.1	Neg

PS736C	Female	Dei	10	122	29	20	-2.6	1.15	0	0	6.18	Neg
PS541C	Female	Alwi	11	129	25	18	-2.41	-1.2	0	0	4.98	Neg
PS535C	Female	Alwi	13	162	48	27	0.81	-0.21	0	0	4.12	Neg
PS549C	Female	Alwi	11	138	26	21	-1.93	-2.57	0	0	1.03	Neg
PS536C	Female	Alwi	10	145	30	22	0.99	-1.39	0	0	0.31	Neg
PS974T	Male	Kayonga	11	143	36	22	-0.02	0.34	0.5	0	0	Neg
PS527C	Female	Alwi	14	160	38	22	0.03	-2.4	0	0	0	Neg
PS557C	Male	Alwi	14	143	35	21	-2.62	-0.94	0	0	0	Neg
PS569C	Male	Alwi	13	144	31	21	-1.62	-1.99	0	0	0	Neg
PS529C	Female	Alwi	12	140	27	19	-1.64	-2.47	0	0	0	Neg
PS961C	Male	Kayonga	11	133	29	19	-1.5	-0.3	0	1	0	Neg
PS714C	Male	Dei	10	112	18	14.2	-4.04	-1.45	0	0	0	Neg
