# Supplementary material for: Transcriptome analysis of peripheral blood of Schistosoma mansoni infected children from the Albert Nile region in Uganda reveals genes implicated in fibrosis pathology
Source: PLoS Negl Trop Dis. 2023 Nov 15;17(11):e0011455. doi: 10.1371/journal.pntd.0011455 (PMC10686515; doi:10.1371/journal.pntd.0011455)
Supplement: S2 Table — (PDF) [file pntd.0011455.s004.pdf]

S2 Table: Significant DEGs between children with high *S. mansoni* infection intensity compared to those with low infection intensity

Comparison	**GeneName**	**log2FoldChange**	**pvalue**	**padj**	**GeneType**	**GeneDescription**
High vs low	CCDC168	3.321	6.38E-06	0.003	protein_coding	coiled-coil domain containing 168
	AC005703.6	1.933	2.30E-06	0.002	TEC	novel transcript
	LYPD8	1.639	0.00147792	0.040	protein_coding	LY6/PLAUR domain containing 8
	AC008505.1	1.464	0.0001769	0.015	lncRNA	novel transcript
	AL358334.3	1.451	3.62E-07	0.001	lncRNA	novel transcript, antisense to PYGL
	NCF4-AS1	1.377	1.16E-06	0.002	lncRNA	NCF4 antisense RNA 1
	CUEDC1	1.355	0.00028987	0.019	protein_coding	CUE domain containing 1
	AC018552.2	1.350	0.00040215	0.022	lncRNA	novel transcript, antisense to CCDC135 and GPR97
	PRMT7	1.330	0.00018046	0.015	protein_coding	protein arginine methyltransferase 7
	AP003064.1	1.318	2.46E-05	0.006	lncRNA	novel transcript
	GLOD5	1.272	0.00240429	0.050	protein_coding	glyoxalase domain containing 5
	AC021097.1	1.265	0.00017632	0.015	lncRNA	novel transcript, antisense to SMARCD3
	SP110	1.233	0.00048546	0.023	protein_coding	SP110 nuclear body protein
	PCAT7	1.211	1.91E-07	0.001	lncRNA	prostate cancer associated transcript 7
	CCDC86	1.148	0.00084791	0.030	protein_coding	coiled-coil domain containing 86
	WFDC21P	1.141	0.00041477	0.022	transcribed_unitary_pseudogene	WAP four-disulfide core domain 21, pseudogene
	PSMB7	1.086	1.74E-08	0.000	protein_coding	proteasome 20S subunit beta 7
	CCDC141	1.084	0.00091745	0.031	protein_coding	coiled-coil domain containing 141
	AC025278.1	1.075	0.00166842	0.041	lncRNA	novel transcript, antisense to EMR4P
	AL137186.1	1.069	2.40E-05	0.006	lncRNA	novel transcript
	UQCC1	1.068	0.00088223	0.030	protein_coding	ubiquinol-cytochrome c reductase complex assembly factor 1
	OGG1	1.065	4.61E-06	0.002	protein_coding	8-oxoguanine DNA glycosylase
	DUS2	1.047	1.05E-06	0.002	protein_coding	dihydrouridine synthase 2

	AP003031.3	1.046	0.00033627	0.020	lncRNA	novel transcript
	FAM170B-AS1	1.029	6.01E-05	0.009	lncRNA	FAM170B antisense RNA 1
	KIF1B	1.027	5.57E-05	0.009	protein_coding	kinesin family member 1B
	C12orf65	1.027	0.00103422	0.032	protein_coding	chromosome 12 open reading frame 65
	AL049647.1	1.018	0.00115988	0.035	lncRNA	novel transcript
	PDZD8	1.014	0.00119263	0.036	protein_coding	PDZ domain containing 8
	AL132642.1	1.005	0.00062658	0.026	lncRNA	novel transcript, antisense to ASB2
	MAMDC2	-0.830	2.71E-05	0.006	protein_coding	MAM domain containing 2
	LINC00863	-0.864	0.00080127	0.029	lncRNA	long intergenic non-protein coding RNA 863
	MALAT1	-1.033	0.0009614	0.031	lncRNA	metastasis associated lung adenocarcinoma transcript 1
