# Supplementary material for: Transcriptome analysis of peripheral blood of Schistosoma mansoni infected children from the Albert Nile region in Uganda reveals genes implicated in fibrosis pathology
Source: PLoS Negl Trop Dis. 2023 Nov 15;17(11):e0011455. doi: 10.1371/journal.pntd.0011455 (PMC10686515; doi:10.1371/journal.pntd.0011455)
Supplement: S3 Table — (PDF) [file pntd.0011455.s005.pdf]

S3 Table: DEGs that may be associated with fibrosis

Expression Gene	Count Publications	Associated with fibrosis	Pubmed IDs (First 500)
OGG1	28	3	36746968,36620083,35654123,35217024,34573096,32977282,32808374,32419286,32209025,30081786,29133960,28587419,28258190,27815257,26370974,26187872,26095584,24918270,24429287,23347351,23219955,23053977,21873502,21212030,20380827,20140303,20074151,17578862,
ITGA4	8	5	35420633,35082796,34876074,34380701,33899950,28692647,26272747,22292410,
SUZ12	2	2	29453456,26290261,
MALAT1	50	12	36760471,36685103,36508811,36217480,36186445,35726125,35593308,35237178,35145971,35126169,34839365,34745428,34697716,34651657,34638230,34481379,33995063,33454598,33422780,33294829,32865781,32596346,32554864,32438606,32425726,32393784,32315984,32203053,32067273,31871512,31853043,31619581,31513886,31081103,30676324,30534359,30146700,30132842,29881817,29604585,29467431,28993096,28900284,28535533,28444861,26919721,26697839,26435214,19635508,17006932,
TPT1	6	1	35174178,30710682,25622969,24818621,24280521,20041318,
AC037198.2	68	33	36787190,36684608,36565192,36430812,36355119,36161819,36058953,35788904,35774294,35704700,35625754,35558266,35487305,35296614,35237609,34746180,34517882,34425215,34282151,34112765,33920030,33706429,33681892,33681182,33414684,33385343,33376743,33361523,33230477,33186919,32835735,32772798,32383980,31913855,31787950,31640463,31083420,30952646,30542457,30539648,30498788,30489086,30393009,29961094,29844053,29276754,28895052,28807667,27621813,27001065,26691002,26681200,26476349,26272747,26150894,26098215,25995382,25929803,25879570,24140711,23756156,23284772,21860248,21854746,21591998,21118800,18636188,17718906,
UQCC1	1	1	35829866,

BLM	1200	48	36944966,36915092,36906971,36903446,36901840,36895392,36878395,36870972,36870575,36869378,36843927,36841380,36834561,36830999,36827329,36823409,36817136,36815239,36814892,36808971,36807664,36805483,36804541,36804125,36793853,36754276,36744773,36733844,36704943,36696855,36688958,36682482,36680813,36677811,36674533,36655684,36641135,36625471,36611939,36610143,36604916,36594067,36586643,36585288,36584920,36562703,36552670,36549352,36536095,36528079,36500388,36474298,36465941,36463728,36461602,36435837,36435270,36433932,36430808,36410185,36410121,36398675,36386126,36371191,36359778,36359232,36356715,36347425,36334818,36333557,36330909,36308405,36304232,36302319,36275905,36267283,36252483,36249808,36228366,36227678,36207753,36176913,36129984,36128650,36111628,36087614,36087509,36082495,36077067,36076570,36075140,36062876,36059455,36050717,36035217,36033388,36003317,35964659,35961069,35954241,35940987,35934841,35932616,35913242,35909199,35903328,35899478,35897764,35887292,35872259,35836804,35836260,35834630,35832075,35822677,35816024,35798357,35794782,35789496,35785413,35784749,35783517,35772783,35747752,35735105,35733864,35733628,35733188,35719213,35716765,35710078,35704641,35702802,35686918,35685407,35668944,35662697,35662628,35661695,35660351,35654123,35636582,35636171,35630905,35628193,35628104,35621164,35617946,35604029,35596212,35566097,35510614,35509835,35472619,35459189,35450342,35435119,35431964,35427207,35420997,35359847,35355866,35355726,35355710,35339032,35328736,35326173,35319169,35291918,35283110,35277778,35268069,35249190,35187969,35178456,35172837,35169250,35149532,35136056,35112775,35086828,35083881,35083738,35083615,35059138,35048404,35013220,35002722,34995795,34990237,34988855,34978427,34977500,34953919,34936986,34890730,34864537,34864115,34852311,34831433,34821087,34803671,34783198,34767797,34758440,34753707,34748761,34746180,34733976,34724865,34692765,34658873,34656907,34643929,34634687,34630661,34630088,34607006,34606948,34600594,34575121,34573030,34563177,34560978,34560512,34536758,34531421,34530920,34529707,34527170,34515769,34484196,34483252,34450310,34445632,34445094,34439906,34427348,34425168,34420515,34419736,34400514,34378791,34375009,34374245,34349830,34349652,34285714,34278915,34276664,34268414,34268377,34243624,34217047,34187349,34175430,34168562,34164493,34123867,34121240,34120115,34118645,34111558,34109242,34098687,34093874,34090522,34061990,34049947,34028626,34022711,34007201,34002012,33995084,33994367,33987261,33981019,33964960,33955458,33955200,33953778,33953686,33952237,33941248,33940030,33912053,33908025,33901873,33892300,33881353,33864298,33861690,33845892,33835703,33808650,33774314,33771973,33760118,33746598,33735625,33716736,33679758,33670189,33654217,33580818,33580396,33576062,33563204,33549109,33549106,33517849,33516805,33512266,33451905,33450497,33391530,33381023,33369271,33340573,33325762,33318630,33293795,33285108,33277324,33259780,33256831,33238752,33202282,33188176,33176882,33174021,33169236,33148433,33144678,33127949,33127511,33

			121292,33090319,33071154,33059466,33045575,33020583,32981275,32973506,32937976,32927253,32924642,32918977,32915635,32912432,32908556,32894569,32884941,32851725,32850975,32848143,32848140,32843954,32828905,32799214,32792953,32781391,32766584,32762376,32758941,32746702,32710728,32663670,32658336,32656894,32630825,32630813,32619930,32618472,32601321,32584210,32571632,32548952,32535538,32535102,32534005,32506869,32474387,32453709,36119009,32448163,32428667,32423816,32390869,32377772,32372165,32362157,32352211,32350681,32323806,32281701,32276127,32269725,32242386,32233122,32192225,32184320,32169098,32145591,32139663,32106023,32075634,32057902,32053892,32033616,32032580,32024440,32019905,32017927,32010478,32002364,32000779,31953203,31949877,31940650,31934168,31907997,31888585,31885648,31884830,31879190,31874367,31866272,31851533,31842999,31840939,31826982,31824505,31820686,31809714,31798444,31786302,31777533,31775746,31757863,31753588,31708243,31706003,31695321,31646730,31646446,31611754,31608748,31608667,31603744,31588241,31555349,31553994,31535412,31505074,31501415,31481954,31473797,31471392,31465731,31452411,31422500,31421181,31419911,31407409,31401393,31354856,31352786,31344551,31329322,31323300,31318036,31302203,31291549,31271423,31256436,31229901,31210057,31176171,31173221,31162951,31155950,31129606,31125560,31115492,31108104,31105564,31100425,31072167,31059283,31049028,31011925,30988156,30928090,30918525,30903918,30890932,30889486,30883698,30870716,

PGD	152	2	36788645,36639317,36627599,36480657,36423200,36004136,35972786,35913788,35911676,35849204,35354317,35344271,35068281,34932185,34412753,34186034,34144220,34030200,33848359,33842976,33755837,33637413,33595729,33500731,33046417,32750037,31676344,31247991,30879544,30696699,30665511,30661024,30657612,30617673,30592952,30171789,30133792,30074834,30062216,29891245,29804404,29479522,29368589,29348826,28787268,28524034,28224801,27992456,27916030,26530270,26493493,26014425,25877792,25687497,25282962,25098029,25016922,24777348,24762087,24658073,24400993,26237262,24163362,23900745,23876679,23856218,23791427,23622646,23523379,23508264,23468849,23378603,23375322,23284823,23276655,25110577,23150080,22997255,22822025,22481026,22407739,22345188,22306439,22241010,21883907,21875427,21692923,21665490,21616676,21403624,21395082,21349925,20864607,20809319,20719937,20692207,20627626,20594975,20160438,19888997,19671582,19439290,19239481,19202707,18937943,18523000,18361812,18174545,17498059,17481912,17449416,17440499,16284066,15908456,15820046,15638197,15322224,15300750,15003814,14759297,14556832,12913874,12900515,12802047,12762414,12470352,12215331,12200467,12116320,12087085,12032730,11866237,11778356,11776197,11601119,11576725,11574669,11228252,11173952,10913957,10775641,10660962,10028321,9949440,9853877,9441871,9238682,8688597,8688580,8650124,21374522,8055673,
KIF1B	2	1	30289982,26255971,
