# Supplementary material for: Transcriptome analysis of peripheral blood of Schistosoma mansoni infected children from the Albert Nile region in Uganda reveals genes implicated in fibrosis pathology
Source: PLoS Negl Trop Dis. 2023 Nov 15;17(11):e0011455. doi: 10.1371/journal.pntd.0011455 (PMC10686515; doi:10.1371/journal.pntd.0011455)
Supplement: S4 Table — (PDF) [file pntd.0011455.s006.pdf]

S4 Table: DEGs associated with TGFB1

Expression Gene	Count Publications	PubMed Ids
BLM	48	36949656,36944966,36082495,35427207,35149532,35013220,34890730,34515769,34439906,33940030,33864298,33277324,32917858,32894569,32884941,32848140,32377772,32233122,32017927,31953203,31907997,31840939,31701721,31535412,30988156,30709583,29971947,29943845,29926178,29642520,29079188,28860147,28752966,27154762,27153807,26777519,24769130,24028731,23980366,23327706,22486844,20945375,16648243,16445573,16415276,15770542,15059909,14694243,
THBS1	33	36193307,35296614,34975404,34854257,34509740,33746571,33414684,32993645,32157392,31913855,31710666,31587040,30880778,30850588,30635789,30489086,29961094,29724129,29228113,28808111,26850833,26658711,26150894,24927163,23850146,23388410,21541268,21118800,19998449,19672301,16809437,16270194,14557872,
MALAT1	12	36852184,35433477,34769245,34326372,32203053,32067273,31432173,30635938,30154407,30146700,27565324,17006932,
ITGA4	5	30687812,28886030,28807052,22292410,22244633,
OGG1	3	32808374,31924810,25740678,
PGD	2	25276836,8104872,
SUZ12	2	26505792,26420484,
A1CF	1	31022160,
KIF1B	1	31935498,
TPT1	1	35197550,
UQCC1	1	32244273,
