# Supplementary material for: Transcriptome analysis of peripheral blood of Schistosoma mansoni infected children from the Albert Nile region in Uganda reveals genes implicated in fibrosis pathology
Source: PLoS Negl Trop Dis. 2023 Nov 15;17(11):e0011455. doi: 10.1371/journal.pntd.0011455 (PMC10686515; doi:10.1371/journal.pntd.0011455)
Supplement: S5 Table — (PDF) [file pntd.0011455.s007.pdf]

S5 Table: Expressed genes in stunting and BMI

GeneName	log2 FoldChange	pvalue	padj	Up/Down	Gene Description	Phenotype
NCEH1	1.281	1.81E-06	0.031	Up	neutral cholesterol ester hydrolase 1	Stunting
MUC5B	1.103	2.00E-06	0.0299	Up	mucin 5B, oligomeric mucus/gel-forming	BMI
DMD	1.139	1.33E-05	0.042	Up	dystrophin	BMI
REXO1L1P	1.300	1.42E-05	0.043	Up	REXO1 like 1, pseudogene	BMI
SERPINA10	-1.127	3.46E-06	0.0299	Down	serpin family A member 10	BMI
