# Supplementary material for: Transcriptome analysis of peripheral blood of Schistosoma mansoni infected children from the Albert Nile region in Uganda reveals genes implicated in fibrosis pathology
Source: PLoS Negl Trop Dis. 2023 Nov 15;17(11):e0011455. doi: 10.1371/journal.pntd.0011455 (PMC10686515; doi:10.1371/journal.pntd.0011455)
Supplement: S6 Table — (PDF) [file pntd.0011455.s008.pdf]

S6 Table: Cell types that differ in relative abundance between *S. mansoni* infected and uninfected children

Cell type	P-value (T-test)	Difference (IU)
MPP	0.012438795	-0.013754584
erythrocytes	0.059665337	-0.06188862
Plasma cells	0.111802411	0.005298362
hMDP	0.122909261	0.015039638
