# Supplementary material for: Transcriptome analysis of peripheral blood of Schistosoma mansoni infected children from the Albert Nile region in Uganda reveals genes implicated in fibrosis pathology
Source: PLoS Negl Trop Dis. 2023 Nov 15;17(11):e0011455. doi: 10.1371/journal.pntd.0011455 (PMC10686515; doi:10.1371/journal.pntd.0011455)
Supplement: S1 Data Analysis Script — (PDF) [file pntd.0011455.s009.pdf]

S_Data Analysis Script: RNA Sequence data

```
setwd("~/Desktop/Albert_finalRNAseq")

#if (!requireNamespace("BiocManager", quietly=TRUE))
  install.packages("BiocManager")
  if (!require("BiocManager"))
  install.packages("BiocManager")
  BiocManager::install("bigPint")
#BiocManager::install("DESeq2")
#BiocManager::install("pcaExplorer")
library(pcaExplorer)
library(DESeq2)

directory<- "~/Desktop/Albert_finalRNAseq"

#Name of file with Filename, sample name, phenotype, sex as attached example

#phenoFile = "All_CAA_CasCon.txt"
#phenoFile = "HAZ_pheno.txt"
#phenoFile = "BMI_pheno.txt"
phenoFile = "ALLcascon.txt"
#phenoFile = "ALLcascon_HC.txt"
#phenoFile = "ALLcascon_HL.txt"
#phenoFile = "ALLcascon_LC.txt

#Get files for each phenotype into lists
#load the phenotype file
phenodata = as.data.frame(read.table(phenoFile, header=TRUE, sep ="\t"))
phenodata$Sex = as.factor(phenodata$Sex)
#phenodata$Age = as.factor(phenodata$Age)
phenodata$Pheno = as.factor(phenodata$Pheno.CAA.)

#create data frame of files
sampleTable <- data.frame(sampleName = phenodata$Sample,
                          fileName = phenodata$File,
                          pheno = phenodata$Pheno.CAA.,
                          #BMI = phenodata$BAZ,
                          #stunt = phenodata$HAZ,
                          sex = phenodata$Sex,
                          age = factor(cut(phenodata$Age, breaks = 3, labels = FALSE)))
                          #stg = phenodata$Stunt.grp)
                          #stunt.grp = factor(cut(phenodata$HAZ, breaks = 3, labels = FALSE)),
                          #BMI.grp = factor(cut(phenodata$BAZ, breaks = 3, labels = FALSE)))
                          #CAA = (cut (log(phenodata$CAA + 1), breaks = 3, labels = FALSE)))
                          #Split log P. falciparum infection into 3 equal size groups.
                          #Pfa = factor(cut(log(phenodata$Pfa), breaks = 3, labels = FALSE)))

#sampleTable$pheno <- factor(sampleTable$pheno)
#Check structure of sampleTable
str(sampleTable)
```

```
#create a DESeq data set (dds) from data frame
ddsHTSeq <- DESeqDataSetFromHTSeqCount(sampleTable = sampleTable,
                                directory = directory,
                                design = ~ pheno + sex + age)

ddsHTSeq <- DESeqDataSetFromHTSeqCount(sampleTable = sampleTable,
                                directory = directory,
                                design = ~ BMI + sex)

ddsHTSeq <- DESeqDataSetFromHTSeqCount(sampleTable = sampleTable,
                                directory = directory,
                                design = ~ stunt + sex)
#Check structure of Dataset
str(ddsHTSeq)


ddsHTSeq
#Filter out genes with minimal data
keep <- rowSums(counts(ddsHTSeq)) >= 100
ddsHTSeq <- ddsHTSeq[keep,]
summary(counts(ddsHTSeq))

#run DESeq to format the data for subsequent analysis
dds<-DESeq(ddsHTSeq)
table(keep)

#get log transformed data for pcaExplorer
rld <- rlog(dds, blind=FALSE)

#Run pcaExporer to visualise data
pcaExplorer(dds = dds, dst = rld)

hist(log(phenodata$HAZ))

#Get differential expression by infection intensity
resCasCon <- results(dds, contrast=c("pheno","CAS","CON"))
resCasConHC <- results(dds, contrast=c("pheno","High","Neg"))
resCasConHL <- results(dds, contrast=c("pheno","High","Low"))
resCasConLC <- results(dds, contrast=c("pheno","Low","Neg"))

#Get differential expression associated with BMI
resBMI <- results(dds,name = "BMI")

#Get differential expression associated with Stunting
resStunt <- results(dds,name = "stunt")

#Load gene annotation data
#EnsGeneNames <- read.delim("~/Documents/TrypanoGEN/rnaseq/index/EnsGeneNames.2.txt", header=TRUE,
row.names=1)
```

```
EnsGeneNames <- read.delim ("~/Desktop/Albert_finalRNAseq/EnsGeneNames.2.txt", header=TRUE, row.names=1)

resCasCon$GeneName = EnsGeneNames$GeneName[match(rownames(resCasCon),rownames(EnsGeneNames))]
resCasCon$GeneType = EnsGeneNames$GeneType[match(rownames(resCasCon),rownames(EnsGeneNames))]
resCasCon$GeneDescription = EnsGeneNames$GeneDescription[match(rownames(resCasCon),rownames(EnsGeneNames))]
resCasCon<-resCasCon[order(resCasCon$padj),]
table(resCasCon$padj <0.05)
table(resCasCon$pvalue <0.05)
table(resCasCon$log2FoldChange >1)
write.csv(file="14.0.23.IU.csv ",resCasCon)

resCasConHC$GeneName = EnsGeneNames$GeneName[match(rownames(resCasConHC),rownames(EnsGeneNames))]
resCasConHC$GeneType = EnsGeneNames$GeneType[match(rownames(resCasConHC),rownames(EnsGeneNames))]
resCasConHC$GeneDescription = EnsGeneNames$GeneDescription[match(rownames(resCasConHC),rownames(EnsGeneNames))]
resCasConHC<-resCasConHC[order(resCasConHC$padj),]
table(resCasConHC$padj <0.05)
table(resCasConHC$pvalue <0.05)
write.csv(file=" read.csv("14.03.23_HC.csv",resCasConHC)

resCasConHL$GeneName = EnsGeneNames$GeneName[match(rownames(resCasConHL),rownames(EnsGeneNames))]
resCasConHL$GeneType = EnsGeneNames$GeneType[match(rownames(resCasConHL),rownames(EnsGeneNames))]
resCasConHL$GeneDescription = EnsGeneNames$GeneDescription[match(rownames(resCasConHL),rownames(EnsGeneNames))]
resCasConHL<-resCasConHL[order(resCasConHL$padj),]
table(resCasConHL$padj <0.05)
table(resCasConHL$pvalue <0.05)
write.csv(file="14.03.23_HL.csv",resCasConHL)

resCasConLC$GeneName = EnsGeneNames$GeneName[match(rownames(resCasConLC),rownames(EnsGeneNames))]
resCasConLC$GeneType = EnsGeneNames$GeneType[match(rownames(resCasConLC),rownames(EnsGeneNames))]
resCasConLC$GeneDescription = EnsGeneNames$GeneDescription[match(rownames(resCasConLC),rownames(EnsGeneNames))]
resCasConLC<-resCasConLC[order(resCasConLC$padj),]
table(resCasConLC$padj <0.05)
table(resCasConLC$pvalue <0.05)
write.csv(file="14.03.23_LC.csv ",resCasConLC)
resBMI$GeneName = EnsGeneNames$GeneName[match(rownames(resBMI),rownames(EnsGeneNames))]
resBMI$GeneType = EnsGeneNames$GeneType[match(rownames(resBMI),rownames(EnsGeneNames))]
resBMI$GeneDescription = EnsGeneNames$GeneDescription[match(rownames(resBMI),rownames(EnsGeneNames))]
resBMI<-resBMI[order(resBMI$padj),]
```

```
  table(resBMI$padj <0.05)
  table(resBMI$pvalue <0.05)
  table(resBMI$log2FoldChange >1)
  write.csv(file="04.01.2023.Final_Albertine.BMI.csv",resBMI)

  resStunt$GeneName = EnsGeneNames$GeneName[match(rownames(resStunt),rownames(EnsGeneNames))]
  resStunt$GeneType = EnsGeneNames$GeneType[match(rownames(resStunt),rownames(EnsGeneNames))]
  resStunt$GeneDescription =
  EnsGeneNames$GeneDescription[match(rownames(resStunt),rownames(EnsGeneNames))]
  resStunt<-resStunt[order(resStunt$padj),]
  table(resStunt$padj <0.05)
  table(resStunt$pvalue <0.05)
  table(resStunt$log2FoldChange >1)
  write.csv(file="13.03.23_Stuntsex.csv",resStunt)

#making volcano plot of DE genes
#BiocManager::install('EnhancedVolcano')

library(EnhancedVolcano)
library(org.Hs.eg.db)

resCasCon = read.csv("14.0.23.IU.csv", row.names = 1)
ens <- rownames(resCasCon)
symbols <- mapIds(org.Hs.eg.db, keys = ens,
            column = c('SYMBOL'), keytype = 'ENSEMBL')
symbols <- symbols[match(rownames(resCasCon), names(symbols))]
VolCasCon = EnhancedVolcano(resCasCon,
          lab = symbols,
          x = 'log2FoldChange',
          y = 'pvalue',
          axisLabSize = 8,
          title = '',
          titleLabSize = 12,
          subtitle = '',
          pCutoff = 0.000104643,
          FCcutoff = 1.0,
          pointSize = 0.5,
          labSize = 3.0,
          col=c('black', 'blue', 'light green', 'red3'),
          #boxedLabels = TRUE,
          colAlpha = 1,
          legendLabels=c('Not sig.','Log2FC','p-value',
          'p-value & Log2FC'),
          legendPosition = 'left',
          legendLabSize = 8,
          legendIconSize = 2.0)
VolCasCon

resCasConHC = read.csv("14.03.23_HC.csv", row.names = 1)
ens <- rownames(resCasConHC)
symbols <- mapIds(org.Hs.eg.db, keys = ens,
```

```
              column = c('SYMBOL'), keytype = 'ENSEMBL')
symbols <- symbols[match(rownames(resCasConHC), names(symbols))]
VolCasConHC = EnhancedVolcano(resCasConHC,
            lab = symbols,
            x = 'log2FoldChange',
            y = 'pvalue',
            axisLabSize = 8,
            title = '',
            titleLabSize = 12,
            subtitle = '',
            pCutoff = 0.0012653,
            FCcutoff = 1.0,
            pointSize = 0.5,
            labSize = 3.0,
            col=c('black', 'blue', 'light green', 'red3'),
            colAlpha = 1,
            legendLabels=c('Not sig.','Log2FC','p-value',
           'p-value & Log2FC'),
            legendPosition = 'right',
            legendLabSize = 8,
            legendIconSize = 2.0)
VolCasConHC
```

resCasConHL = read.csv("14.03.23_HL.csv", row.names = 1)

```
ens <- rownames(resCasConHL)
symbols <- mapIds(org.Hs.eg.db, keys = ens,
              column = c('SYMBOL'), keytype = 'ENSEMBL')
symbols <- symbols[match(rownames(resCasConHL), names(symbols))]

VolCasConHL = EnhancedVolcano(resCasConHL,
            lab = symbols,
            x = 'log2FoldChange',
            y = 'pvalue',
            axisLabSize = 8,
            title = '',
            titleLabSize = 12,
            subtitle = '',
            pCutoff = 0.00240429,
            FCcutoff = 1.0,
            pointSize = 0.5,
            labSize = 3.0,
            col=c('black', 'blue', 'light green', 'red3'),
            colAlpha = 1,
            legendLabels=c('Not sig.','Log2FC','p-value',
                     'p-value & Log2FC'),
            legendPosition = 'left',
            legendLabSize = 8,
            legendIconSize = 2.0)
VolCasConHL
```

resCasConLC = read.csv("14.03.23_LC.csv", row.names = 1)

```
ens <- rownames(resCasConLC)
symbols <- mapIds(org.Hs.eg.db, keys = ens,
          column = c('SYMBOL'), keytype = 'ENSEMBL')
symbols <- symbols[match(rownames(resCasConLC), names(symbols))]

VolCasConLC = EnhancedVolcano(resCasConLC,
        lab = symbols,
        x = 'log2FoldChange',
        y = 'pvalue',
        axisLabSize = 8,
        title = '',
        titleLabSize = 12,
        subtitle = '',
        pCutoff = 0.0001,
        FCcutoff = 1.0,
        pointSize = 0.5,
        labSize = 3.0,
        col=c('black', 'blue', 'light green', 'red3'),
        colAlpha = 1,
        legendLabels=c('Not sig.','Log2FC','p-value',
               'p-value & Log2FC'),
        legendPosition = 'right',
        legendLabSize = 8,
        legendIconSize = 2.0)
VolCasConLC



install.packages("cowplot")
library(cowplot)
plot_grid(VolCasCon,VolCasConHC,VolCasConHL,VolCasConLC, nrow = 2, labels = c("A","B", "C", "D"))
resStunt = read.csv("13.03.23_Stuntsex.csv", row.names = 1)
ens <- rownames(resStunt)
symbols <- mapIds(org.Hs.eg.db, keys = ens,
          column = c('SYMBOL'), keytype = 'ENSEMBL')
symbols <- symbols[match(rownames(resStunt), names(symbols))]

VolresStunt = EnhancedVolcano(resStunt,
        lab = symbols,
        x = 'log2FoldChange',
        y = 'pvalue',
        axisLabSize = 10,
        title = '',
        titleLabSize = 12,
        pCutoff = 1.91E-06,
        FCcutoff = 1.0,
        pointSize = 2.0,
        labSize = 3.0,
        col=c('black', 'blue', 'light green', 'red3'),
        colAlpha = 1,
        legendLabels=c('Not sig.','Log2FC','p-value',
```

```
                    'p-value & Log2FC'),
            legendPosition = 'right',
            legendLabSize = 12,
            legendIconSize = 4.0)
VolresStunt

resBMI = read.csv("Final_Albertine.BMI.csv", row.names = 1)
ens <- rownames(resBMI)
symbols <- mapIds(org.Hs.eg.db, keys = ens,
              column = c('SYMBOL'), keytype = 'ENSEMBL')
symbols <- symbols[match(rownames(resBMI), names(symbols))]

VolresBMI = EnhancedVolcano(resBMI,
            lab = symbols,
            x = 'log2FoldChange',
            y = 'pvalue',
            axisLabSize = 10,
            title = '',
            titleLabSize = 12,
            pCutoff = 1.44E-05,
            FCcutoff = 1.10,
            pointSize = 2.0,
            labSize = 3.0,
            col=c('black', 'blue', 'light green', 'red3'),
            colAlpha = 1,
            legendLabels=c('Not sig.','Log2FC','p-value',
                    'p-value & Log2FC'),
            legendPosition = 'right',
            legendLabSize = 12,
            legendIconSize = 4.0)
VolresBMI

plot_grid(VolresStunt,VolresBMI, nrow = 2, labels = c("A","B"))

##########################################################################################
```
