# Supplementary material for: Transcriptome analysis of peripheral blood of Schistosoma mansoni infected children from the Albert Nile region in Uganda reveals genes implicated in fibrosis pathology
Source: PLoS Negl Trop Dis. 2023 Nov 15;17(11):e0011455. doi: 10.1371/journal.pntd.0011455 (PMC10686515; doi:10.1371/journal.pntd.0011455)
Supplement: Attachment — Submitted filename: Response_to_reviewers.docx [file pntd.0011455.s010.docx]

Dear Dr Mulindwa,
 
Thank you very much for submitting your manuscript "Transcriptome analysis of peripheral blood of Schistosoma mansoni infected children from the Albert Nile region in Uganda reveals genes implicated in fibrosis pathology." for consideration at PLOS Neglected Tropical Diseases. As with all papers reviewed by the journal, your manuscript was reviewed by members of the editorial board and by several independent reviewers. In light of the reviews (below this email), we would like to invite the resubmission of a significantly-revised version that takes into account the reviewers' comments.
 
This is an interesting manuscript. Please consider the reviewers' thorough comments included, particularly the question about whether the pathways analysis may be revised or removed. It would also be helpful if the authors can provide further information to clarify the study design and the decisions to include or exclude some samples.

We cannot make any decision about publication until we have seen the revised manuscript and your response to the reviewers' comments. Your revised manuscript is also likely to be sent to reviewers for further evaluation.
 
When you are ready to resubmit, please upload the following:

[1] A letter containing a detailed list of your responses to the review comments and a description of the changes you have made in the manuscript. Please note while forming your response, if your article is accepted, you may have the opportunity to make the peer review history publicly available. The record will include editor decision letters (with reviews) and your responses to reviewer comments. If eligible, we will contact you to opt in or out.

[2] Two versions of the revised manuscript: one with either highlights or tracked changes denoting where the text has been changed; the other a clean version (uploaded as the manuscript file).

Important additional instructions are given below your reviewer comments.

Please prepare and submit your revised manuscript within 60 days. If you anticipate any delay, please let us know the expected resubmission date by replying to this email. Please note that revised manuscripts received after the 60-day due date may require evaluation and peer review similar to newly submitted manuscripts.
 
Thank you again for your submission. We hope that our editorial process has been constructive so far, and we welcome your feedback at any time. Please don't hesitate to contact us if you have any questions or comments.
 
Sincerely,

Jennifer A. Downs, M.D., Ph.D.
Academic Editor
PLOS Neglected Tropical Diseases

Eva Clark
Section Editor
PLOS Neglected Tropical Diseases

***********************

This is an interesting manuscript. Please consider the reviewers' thorough comments included, particularly the question about whether the pathways analysis may be revised or removed. It would also be helpful if the authors can provide further information to clarify the study design and the decisions to include or exclude some samples.


Reviewer's Responses to Questions

<b>Key Review Criteria Required for Acceptance?</b></br></br>
As you describe the new analyses required for acceptance, please consider the following:</br></br>

<b>Methods</b></br></br>
-Are the objectives of the study clearly articulated with a clear testable hypothesis stated?</br>
-Is the study design appropriate to address the stated objectives?</br>
-Is the population clearly described and appropriate for the hypothesis being tested?</br>
-Is the sample size sufficient to ensure adequate power to address the hypothesis being tested?</br>
-Were correct statistical analysis used to support conclusions?</br>
-Are there concerns about ethical or regulatory requirements being met?</br></br>

Reviewer #1: The authors have provided detailed description of the experiments' methodology.

Reviewer #2: 1. Major: I would like more information about the study. Was screening done as part of a public health effort  and then a subset were asked to participate in a research study?

We thank the reviewer for this question. This was a cross-sectional study which involved screening for *S. mansoni* and a subset of the participants who consented were selected to participate in the study. We have clarified this by updating line 113 – 115 in the manuscript as:

The gene expression study was part of a cross sectional study conducted between October and November 2020 to determine schistosomiasis prevalence among primary school going children aged between 10 to 15 years.

Recruitment was after receiving signed consent and assent as stated in line 127 – 128

Participants who provided urine for screening were recruited to the study after being interviewed and informed consent signed by the parent/guardian and assent by the child.

When did the research component start – with collection of the screening urine or the blood sample? This could be made clearer in the text and in Figure 1.

The research component started with collection of the blood sample after obtaining informed consent stated in line 127 -128. We have updated this in **Figure 1**.

Were there only 10-15 year olds in the screening?

Yes only 10 -15-year-olds were screened. We have updated this in the screening statement in the manuscript line 114 - 115.

Were they only screened for schistosomiasis or for other parasites?  If other parasites, these would be important to include as confounders.

Participants were screened for schistosomiasis and other stool parasites. No other parasites in stool were found in the samples selected for RNAseq. This detail has been included in line 125 – 126 of the manuscript.

Samples were also tested for *S. mansoni* and other parasites eggs in stool using Kato Katz (KK) (**Table S1**) as earlier published.

The ethics statement includes stool examination for eggs but I didn’t see egg data included for your study participants – were children with positive POC-CCA also treated with praziquantel?

We have included the egg data for the RNAseq study participants in the supplementary data **Table S1**.

Children positive with POC-CCA were treated with praziquantel (40mg/kg dosage) as indicated in the Ethics statement line 103 – 105.

If the TrypanoGEN+ study protocol has been published, please reference it as this could clear up a lot of these questions. In the results, there were 727 in “further studies” and 152 in the “gene expression study based on POC-CCA scores.” What was it about the POC-CCA that led those 152 participants to be recruited for this study?

The participants for the gene expression study were purposively selected based on their POC CCA scores in order to categorise them by infection intensity. POC CCA testing is quick (less than 20 min) and this enabled collection of Paxgene blood from the participants. CAA was later done on the plasma from the same participants to ascertain their infection intensity, which was subsequently used as the phenotype in the differential gene expression analysis. We have included this in line 123 – 124:

Selection for inclusion in the RNAseq study was purposively done based on the POC-CCA scores.

and line 133 – 134

POC-CCA was used in the field to classify participants by infection intensity to collect blood samples that were sequenced for RNAseq.

2.      Major: CAA can be positive for Schistosoma haematobium and/or S. mansoni. If the Albert-Nile area is endemic only for S. mansoni, I would include that information. If it is endemic for both, did you confirm that eggs were mansoni rather than haematobium?

Thank you for this suggestion. We have added literature of the main cause of schistosomiasis in Uganda on line 62 -64.

Schistosomiasis in Uganda has been shown to be mainly caused by *Schistosoma mansoni* [1,2] with low pockets of *Schistosoma haematobium*[3,4].

Although it is rare, *S. mansoni* can cause urogenital schistosomiasis. Did the participants have urine and stool microscopy to assess for eggs?

The participants only had stool done microscopy to assess for eggs but not urine microscopy.

3.      Major: Blood collected in a PAXgene tube usually has RNA extracted using the Paxgene kit, but the authors used Trizol. I am unable to find a published study validating Trizol for RNA extraction from Paxgene tubes, and the reference given (#11) does not specify the RNA extraction method for blood collected in Paxgene tubes. Can the authors elaborate on the rationale for this approach, ideally with a reference validating this method?

The reference is a protocol and highlights the process of extracting RNA from PAXgene blood using Trizol. We have also added another reference which compared three kits used to extract RNA from PAXgene tubes with Trizol having a higher RNA yield compared to the rest. Line 152 – 153.

4.      Minor: Since your audience is not all Schisto experts, would describe what CAA and CCA are and why you did both and whether or not people’s CAA and CCA results needed to be concordant to be included in the study.

We have included a description for CAA and CCA in the literature in line 69 – 74.

POC-CCA is available in a cassette format to detect the Cathodic Circulating Antigen (CCA) secreted by schistosomes in urine within 20 minutes. It is a qualitative test and has been recommended to be used to screen for *S. mansion* infections in the field. The UCP-LF CAA on the other hand detects the Circulating Anodic Antigen (CAA) in blood and urine. This test detects even the lowest schistosomiasis infection[5] but cannot be conducted in the field as it requires processing in a laboratory setting.

5.      Minor: Give rationale for assigning someone as low vs. high CAAemia – how did you decide on the cutoff?

Assigning CAA classification was based on previously published literature by Tamarozzi et al in 2021. This has been included in line 148 -150.

6.      Minor: Include the details of the POC-CCA used (manufacturer, etc).

This detail has been included in line 118 – 119, Rapid Medical Diagnostics, Pretoria, South Africa, batch No. 191031120.

7.      Minor: Spell out what the UCP-LF of UCP-LF CAA is and include its details (manufacturer, etc).

This detail has been included in line 141– 142, up-converting phosphor lateral flow- circulating Anodic Antigen, (UCP-LF CAA).

8.      Typo: line 123 “CAA concentrations > 30 pg/mL” should be “< 30 pg/mL”

Thank you for this observation, we missed out the “positive”. This has been updated in line 148 – 150 to read:

CAA concentrations > 30 pg/mL were classified as **positive**; negative (CAA < 30 pg/mL), low infection intensity (CAA 30 to <1000 pg/mL) and high infection intensity (CAA > 1000 pg/mL).

9.      Typo: line 128 clarify if it should be “quantity of 1 ug” or a “concentration of 1 ug” per what volume.

The statement has been updated to read:

The RNA was quantified using Qubit (Invitrogen, USA) and samples with >1µg of RNA were shipped to the Centre for Genomics Research at the University of Liverpool for sequencing where the quality of the samples was checked using an Agilent Bioanalyser” from line 153 – 156.

10.     Typo: line 135 For clarity, would specify 2x150 bp and 30 million

We have specified this in the manuscript, now line 160 – 162.

The libraries were sequenced on an Illumina NovaSeq S4 (Illumina) in the 2x150 bp read configuration to a target depth of 30 million read pairs per sample at the Centre for Genomic Research at the University of Liverpool

Reviewer #3: -Are the objectives of the study clearly articulated with a clear testable hypothesis stated? Yes
-Is the study design appropriate to address the stated objectives? Yes
-Is the population clearly described and appropriate for the hypothesis being tested? Yes
-Is the sample size sufficient to ensure adequate power to address the hypothesis being tested? This study is a hypothesis generating type of study. So no problem.
-Were correct statistical analysis used to support conclusions? Yes
-Are there concerns about ethical or regulatory requirements being met? No

--------------------

<b>Results</b></br></br>
-Does the analysis presented match the analysis plan?</br>
-Are the results clearly and completely presented?</br>
-Are the figures (Tables, Images) of sufficient quality for clarity?</br></br>

Reviewer #1: The results are clearly.

Reviewer #2: 1. Major: 80 samples were “selected for sequencing to represent the extremes of infection intensity” which I assumed meant their CAA-determined infection intensity, yet “11 lacked CAA results and were excluded.” Could the authors elaborate on this?

Samples were selected for RNAseq based on CCA, 152 children who had extreme CCA scores (values) had blood collected in PAXgene tubes of which 81 had sufficient RNA required by the sequencing laboratory for sequencing. Out of these CAA results were obtained for 64 samples. Analysis was done based on CAA extremes. We have further elaborated this in the manuscript line 189 – 196 as:

Following the screening of 914 children aged between 10 – 15 years in Pakwach District, 727 children were recruited for further studies [6] of which 152 children were recruited to participate in this gene expression by RNAseq study. The samples were selected for RNAseq based on CCA. 152 children with extreme CCA values had blood collected in PAXgene tubes of which eighty-one (81) samples had the required amount of RNA for sequencing recommended by the Sequencing Laboratory. One (1) did not pass QC and was not sequenced. Eighty (80) samples were therefore sequenced to represent the extremes of infection intensity. Of the 80 sequenced, 11 lacked CAA results and were excluded (**Figure 1**).

Additionally, only 52% of RNA passed QC, which seems very low to me, and I am assuming is because of the 1 ug cutoff. Why was the cutoff so high? The amount of input RNA for RNA-Seq is usually much lower than 1 ug. My concern would be that participants with lower WBC counts would be excluded since they wouldn’t generate enough RNA to meet that cutoff which could skew your results.

This was total RNA that was supplied meaning that about 50ng of mRNA was available for sequencing. While there are methods for sequencing with smaller amounts the use of 1ug permits the use of methods that avoid amplification steps that can bias results and was the quantity requested by the Center for Genomic Research in Liverpool that did the sequencing

<https://www.liverpool.ac.uk/genomic-research/technical-resources/>

2.      Major: While it is inconvenient when there are people who do not cluster in a PCA, I’m not sure that excluding 5 people is the right approach, nor do the reasons given seem appropriate. We don’t usually exclude people from analysis because of their ethnicity. Poor RNA quality should have been detected with Qubit and Agilent bioanalyzer QC before sequencing. Which schisto groups were these 5 people in?

We now state that we do not know why the samples did not cluster rather than speculating that ethnicity or sample quality was the problem. The plot showing the outliers (Figure S1) reveals a very high degree of scatter for these five samples relative to the dense cluster of the remaining samples.

Of the 5 samples excluded, 3 were high infection intensity and 2 were negative. We have updated the text in the manuscript line 196– 199 to read:

- PCA analysis identified five samples (3 high infection intensity, 2 negative) which did not cluster closely with the remaining samples (**Figure S1**). As we do not know why these samples did not cluster with the others, we took a conservative approach and excluded them from the analysis.

3.      Major: There are so few differentially expressed genes, and so few that are included in the Reactome database, that I don’t think that pathways analysis is an appropriate approach. There is only 1 gene included in each pathway of Tables 4 and S4 and 1 or 2 genes for each pathway in Table S8. Although the p-value numbers indicate statistical significance, I question if 1 differentially expressed gene is enough to invoke pathologic importance for that pathway. It may be that there really aren’t that many differences in gene expression between the groups and that alone is the conclusion – adding pathways analysis seems like trying to create a difference where there might not be one.

Thank you for this suggestion. We agree with the reviewer and have decided to exclude the pathway analysis.

4.      Major: The BMI and stunting subanalyses are only done in the schistosomiasis group – why is this? In discussion, would need to clarify that the associations are for BMI and stunting in people with schistosomiasis at the time of the study. Were these studies done for a subset of the differentially expressed genes or an analysis of all of the RNASeq data?

Analysis was done for all infected and uninfected. Our heading was ambiguous and has been rephrased to make this clear. “Expression of genes associated with stunting and BMI ” line 232.

5.      Major: The inclusion of the PubMed search results as part of methods and results for specific gene products, while thorough, would seem to be more appropriate for integration into the discussion where there are relevant citations, for example the authors do this in lines 288-290 with reference 28.

Thank you for this suggestion. We have integrated this in the discussion on line 280 – 282

We searched through Pubmed abstracts and titles using each of the 63 unique differentially expressed gene names and the terms “schistosomiasis” or “schistosoma” and fibrosis.

The findings were incorporated in line 286 - 291

None of the 63 genes had informative associations with schistosomiasis following the Pubmed search with the terms “schistosomiasis” or “schistosoma”. The search for abstracts and titles using the term “fibrosis” with each of the 63 genes in turn identified 27 gene names that appeared in articles that also mentioned fibrosis of which 13 had well documented associations with fibrosis (Table S3). Seven genes were also associated with TGFB1 (Table S4).

6.      Minor: How did the authors compensate for the multiple comparisons – 4 between-group comparisons (neg vs pos, high vs low, high vs neg, low vs neg) – in deciding on a cutoff for statistical significance?

Thank you for this question. We had no further statistical correction was done. We have added a sentence in line 175 – 177:

As the objective of the study was hypothesis generating, no additional statistical correction was made to the p-adjusted values produced by DESeq2 for the four comparisons that were analysed.

7.      Minor: Was the regression analysis for differential gene expression only done for the subset of genes already found to be differentially expressed in the S. mansoni group?
Regression analysis for association of BMI and stunting were done on the whole gene expression data. This has been clarified by deleting the “*S. mansoni* infected children” in the heading on line 232 and in the paragraph line 237.

Reviewer #3: -Does the analysis presented match the analysis plan? Yes
-Are the results clearly and completely presented? No
-Are the figures (Tables, Images) of sufficient quality for clarity? No

--------------------

<b>Conclusions</b></br></br>
-Are the conclusions supported by the data presented?</br>
-Are the limitations of analysis clearly described?</br>
-Do the authors discuss how these data can be helpful to advance our understanding of the topic under study?</br>
-Is public health relevance addressed?</br></br>

Reviewer #1: This article provides sufficient information to understand the genetic changes in human children infected with Schistosoma mansoni. The results have potential implications for the diagnosis and treatment of schistosomiasis.

Reviewer #2: Because of the aforementioned questions/concerns about the data analysis approach, it is difficult to assess the conclusions. The limitations noted by this reviewer were not considered in the conclusion as written. While the findings of association of some of the differentially expressed genes in the schistosomiasis group with fibrosis is interesting, there were so few differentially expressed genes (and only 4 protein-coding) that the conclusions may have been overstated.

Reviewer #3: -Are the conclusions supported by the data presented? Yes
-Are the limitations of analysis clearly described? Yes
-Do the authors discuss how these data can be helpful to advance our understanding of the topic under study? Not much, should have mentioned about the benefit for future diagnosis and treatment.
-Is public health relevance addressed? No

--------------------

<b>Editorial and Data Presentation Modifications?</b></br><br/>
Use  this section for editorial suggestions as well as relatively minor modifications of existing data that would enhance clarity. If the only modifications needed are minor and/or editorial, you may wish to recommend “Minor Revision” or “Accept”.

Reviewer #1: Accept

Reviewer #2: 1. Major -- add relevant available demographic data to Table 1 – age, coinfections, BMI, stunting, what subcounty site a person was from, average CAA, % with S. mansoni eggs seen in stool, etc. Any significant differences in these variables should be addressed.

Thank you. Other demographic data has been included and uploaded as supplementary data in **Table S1**

2.      Major – consider if the fold changes, adjusted p-values, and FDR should/need to be taken to the millionths place in Tables 3, 5, S2, S3, S4.

Thank you for this suggestion. We have reduced the decimal places in the tables.

3.      Major – Figures 3 and 4 are illegible (cannot assess contents given small text size and low resolution)

We have updated the figures 3 and 4 (now figure 5).

4.      Please specify where the RNA-Seq data are deposited.

The EGA No. is EGAS00001007173

5.      Consider providing the code used for analysis as supplemental material or a link to a github with that information.

The code has been added in the supplementary material

Reviewer #3: Minor modification is enough.

--------------------

<b>Summary and General Comments</b></br></br>
Use  this section to provide overall comments, discuss strengths/weaknesses of the study, novelty, significance, general execution and scholarship. You may also include additional comments for the author, including concerns about dual publication, research ethics, or publication ethics. If requesting major revision, please articulate the new experiments that are needed.

Reviewer #1: In this manuscript, the authors investigated the RNA sequencing of 44 Schistosoma mansoni-infected children and 20 uninfected children, with the reads aligned to the GRCh38 human genome. The study is well-conducted, and its scientific soundness is reasonable. However, before publication, some points should be addressed, which are discussed below:

1.      It is recommended to reconfirm the RNA expression of significant DEGs (Differentially Expressed Genes) using qPCR, especially for CLTP1, SUZ12, and TRBC2.

Whilst confirmation with qPCR was common in the early days of RNAseq it is no longer routinely used. A search of Pubmed with RNAseq found 45,384 references and with RNAseq and qPCR found 2,940 references. We agree that validation is desirable but believe that this should be done on a new set of samples to test for biological as well as technical reproducibility.

2.      Apart from CLTP1, SUZ12, and TRBC2 genes, more detailed explanations should be provided for other significant DEGs identified in the study.
The DEGs that were identified were run through a Pubmed search included in Table S4. Some of these genes were predicted to reduce fibrosis whereas other to increase it.

Reviewer #2: In this manuscript, Namulondo and colleagues use transcriptomics to compare peripheral blood gene expression in young adolescents with or without schistosomiasis and with low vs. high schistosomal burden. I appreciate the important work to describe the human immune status during schistosomal infection using gene expression studies. The main finding may be that there aren’t many alterations in gene expression in the peripheral blood – in itself, an interesting finding! Namulondo et al. do mention that it may be that the worms don’t induce an immune response, but then go on to do the pathways and other analyses to try to find patterns in 14 differentially expressed genes, of which only 4 were protein encoding, with statistically significant pathways only including one of the differentially expressed genes.  I would recommend focusing on the similarities between schisto/no schisto rather than the differences.
Thank you for this recommendation. We looked at the similarities in gene expression across the comparisons (Figure 4) and have included this in the manuscript line 222 – 230 as:

We observed DEGs that were common in particular comparisons with similar expression and some that were unique to only one comparison (Figure 4). One (1) DEG, CCDC168 was upregulated in the three comparisons (IU, HU and HL). Additionally, 7 DEGs, 5 (SRGAP3-AS2, AC104035.1, CLIP1, LINC0095 and AC005153.1) upregulated and 2 (ZNF217 and SUZ12) downregulated common to the IU and HU comparison while fourteen (13 (GLOD5, AC005703.6, SP110, PRMT7, CCDC141, CCDC86 , AL049647.1, CUEDC1, AC025278.1, FAM170B-AS1, KIF1B, AL132642.1) upregulated and 1 (MALAT1) downregulated) DEGs were observed in HU and HL comparison and none in IU and HL comparisons.

Reviewer #3: This paper is designed to characterize different types of Schistosoma mansoni infected persons in highly endemic area in Uganda by RNAseq analysis using peripheral blood. Density of parasite load was evaluated by circulating antigen (CCA and CAA) levels. High, Low, and No infection groups with stunting and BMI, Blood cell types were well defined. their conclusion that high levels of active infection influenced the expression levels of limited number of genes that were mainly related to fibrotic and TGF-B1 activation. Although the results are simply descriptive, this transcriptome information will be beneficial for future development of relevant research.

There are several comments to be improved.
1. Fig.3 and Fig 4 needs clearer picture with high resolution. And more important is to make more easy-to-understand legends that explain the details of the methodology.

We have updated the figures 3 and 4 (now figure 5). Legends have been included to explain the details in the methodology.

2. Cell types analysis needs more explanation. How the authors identified MPP showed a significant change between the groups?

We added information on this in line 244 – 246.

Bisque analysis uses reference single cell sequence datasets to estimate proportions of different cell types in bulk RNAseq data.

3. There was no description of antibody levels of the individuals. If available, please describe it.

We did not collect antibody data.

4. Generally, fibrosis in the liver is frequently observed in the endemic area? Even younger generation?

Some studies have shown fibrosis of the liver to occur among children between 6 – 20 years (Da Silva LC, Chieffi PP, Carrilho FJ. 2005).

--------------------
PLOS authors have the option to publish the peer review history of their article (<a href="<https://journals.plos.org/plosntds/s/editorial-and-peer-review-process#loc-peer-review-history>" target="_blank">what does this mean?</a>). If published, this will include your full peer review and any attached files.<br><br>

If you choose “no”, your identity will remain anonymous but your review may still be made public.<br><br>

<b>Do you want your identity to be public for this peer review?</b> For information about this choice, including consent withdrawal, please see our <a href="<https://www.plos.org/privacy-policy>" target="_blank">Privacy Policy</a>.

Reviewer #1: No

Reviewer #2: No

Reviewer #3: No


Figure Files:
While revising your submission, please upload your figure files to the Preflight Analysis and Conversion Engine (PACE) digital diagnostic tool, [https://pacev2.apexcovantage.com](https://pacev2.apexcovantage.com/). PACE helps ensure that figures meet PLOS requirements. To use PACE, you must first register as a user. Then, login and navigate to the UPLOAD tab, where you will find detailed instructions on how to use the tool. If you encounter any issues or have any questions when using PACE, please email us at [figures@plos.org](mailto:figures@plos.org).
 
Data Requirements:
Please note that, as a condition of publication, PLOS' data policy requires that you make available all data used to draw the conclusions outlined in your manuscript. Data must be deposited in an appropriate repository, included within the body of the manuscript, or uploaded as supporting information. This includes all numerical values that were used to generate graphs, histograms etc.. For an example see here: <http://www.plosbiology.org/article/info%3Adoi%2F10.1371%2Fjournal.pbio.1001908#s5>.
 
Reproducibility:
To enhance the reproducibility of your results, we recommend that you deposit your laboratory protocols in [protocols.io](http://protocols.io/), where a protocol can be assigned its own identifier (DOI) such that it can be cited independently in the future. Additionally, PLOS ONE offers an option to publish peer-reviewed clinical study protocols. Read more information on sharing protocols at <https://plos.org/protocols?utm_medium=editorial-email&utm_source=authorletters&utm_campaign=protocols>

__________________________________________________
In compliance with data protection regulations, you may request that we remove your personal registration details at any time.  (Use the following URL: <https://www.editorialmanager.com/pntd/login.asp?a=r>). Please contact the publication office if you have any questions.
