# Supplementary material for: Transcriptome analysis of peripheral blood of Schistosoma mansoni infected children from the Albert Nile region in Uganda reveals genes implicated in fibrosis pathology
Source: PLoS Negl Trop Dis. 2023 Nov 15;17(11):e0011455. doi: 10.1371/journal.pntd.0011455 (PMC10686515; doi:10.1371/journal.pntd.0011455)
Supplement: Attachment — Submitted filename: Response_to_review_comments.docx [file pntd.0011455.s011.docx]

**Response to review comments**

The results that no other parasites were found in the stool of the children selected for RNA-Seq is not clearly presented and should be mentioned in the text.

The results of other parasites have now been included in line 194 – 195. The text now reads:

*Schistosoma mansoni* eggs were found in the samples for RNAseq, however no other parasite eggs were detected by KK in these samples (**Table S1**).

The ethics statement states that children who had eggs in stool were treated with praziquantel, but there is still no comment about those who were POC-CCA positive being treated.
The study was done alongside the national control program who are in charge of PZQ administration. The country’s policy bases treatment on eggs in stool and not POC-CCA. We therefore didn’t treat based on POC-CCA results. We have included a sentence in line 105 – 106 that reads:

POC-CCA results were not used as an indication for treatment.

Reference 16 reports CAA cut-offs that are different than what was used in this manuscript. Please reconcile this. Also, in lines 148-150 there is not a clear classification for what would happen if someone’s CAA result was exactly 1000 pg/mL.
Thank you for the comment. We have changed the reference to Corstjens et al which specifies the cut off concentration based on the CAA assay used and as well included guidance by email communication from the Leiden assay development team. We have also updated the classification CAA 30 – 1000pg/mL for low infection intensity. The sentence now in line 149 – 152 therefore reads:

CAA concentrations > 30 pg/mL were classified as positive; negative (CAA < 30 pg/mL), low infection intensity (CAA 30 - 1000 pg/mL) and high infection intensity (CAA > 1000 pg/mL) adapted from Corstjens et al [16] as well as guided by email communication from the Leiden assay development team.

The EGA number does not appear to be included in the text anywhere.

We have included a section on Data availability in line 363 - 366

The discussion does not include a paragraph about limitations of this study, which were pointed out by reviewers. Please add this and be sure to include:
-POC-CCA doesn’t differentiate between species; urine was not filtered.
-Please mention the requirement that samples had to have 1 ug of RNA, which was done to avoid amplification steps but could still introduce bias because of excluding those with lower amounts of RNA.
-Statistical analysis did not correct for multiple comparisons and an additional laboratory method (e.g. qPCR or other method) to validate these findings would be useful.
We added a section on limitations from line 337 – 345. The section reads:

**Limitations of the study**

There were few differentially expressed genes which limited further analysis of pathways affected by the genes. POC-CCA test doesn’t differentiate between species and therefore further testing to rule out S. haematobium may have been an added advantage although this was not done. To avoid amplification steps, only samples with 1 ug of RNA were sequenced although this could still introduce bias due to exclusion of samples with lower amounts of RNA. Additionally, statistical analysis did not correct for multiple comparisons and an additional laboratory method (e.g. qPCR or other method) to validate these findings would be useful.
